# American Medical Association

**Published:** 1852-11

**Authors:** 


*American Medical Association.*—At the meeting of the Association held at Richmond, Va., May, 1852, the undersigned were appointed a committee to receive voluntary communications on medical subjects, and to award two prizes of $100 each to the authors of the best two essays.

Each communication must be accompanied by a sealed packet, containing the name of the author, which will be opened only in the case of the successful competitors. Unsuccessful communications will be returned on application after June 1st, 1853.

Communications must be addressed, post-paid, to the chairman of the committee, Dr. Joseph M. Smith, 56 Bleecker street, New York, on or before the 20th of March, 1853.

Joseph M. Smith, M. D.
John A. Swett, M. D.
W. Parker, M. D.
Gurdon Buck, M. D.
Alfred C. Post, M. D.

New York, September 17, 1852.

Editors of medical journals in the United States are respectfully requested to copy the above.

